# Health system stakeholders’ perceptions of religious and socio-cultural influences on measles vaccine hesitancy in Madagascar: a qualitative study

**DOI:** 10.1186/s12889-026-28130-5

**Published:** 2026-06-15

**Authors:** Félix Alain, Mbolatiana Raharinivo, Mamy Jean Jacques Razafimahatratra, Faly Hariniaina Randrianasolo, Holinirina Ramananjanahary, Mialy Mathieu Andriambelo, Jean Claude Andrianirinarison, Paubert Tsivahiny, Hanitriniala Pâquerette Sahondranirina, Lethicia Lydia Yasmine, Diana Ratsiambakaina

**Affiliations:** 1 Ministry of Public Health, National Institute of Public and Community Health (INSPC), Antananarivo, 101 Madagascar; 2 Faculty of Medicine, University of Mahajanga, Mahajanga, 401 Madagascar; 3 Faculty of Medicine, University of Antananarivo, Antananarivo, 101 Madagascar; 4 Expanded Programme On Immunisation Directorate (EPID), Ministry of Public Health, Antananarivo, 101 Madagascar; 5 Tropical Institute of Odonto-Stomatology of Madagascar (IOSTM), Mahajanga, 401 Madagascar

**Keywords:** Measles, Vaccination, Madagascar, Religious beliefs, Socio-cultural barriers, Qualitative study

## Abstract

**Background:**

In Madagascar, measles remains a leading cause of infant mortality, and recurrent epidemics highlight the urgent need to strengthen vaccination strategies. Despite regular mass immunisation efforts, such as the national campaign in October 2024, the effectiveness of these programmes is often undermined by complex non-medical factors. This study aims to qualitatively explore the specific religious and socio-cultural beliefs and perceptions that act as barriers to the acceptance of the measles vaccine across the country.

**Methods:**

This qualitative exploratory study was integrated into the 2024 Post-Measles Campaign Evaluation conducted across all 23 regions of Madagascar. We used purposive sampling to conduct semi-structured, in-depth interviews with key health system stakeholders, including regional health officials, community leaders, and managers of Basic Health Centres. Data were analysed using manual inductive thematic content analysis to identify recurring patterns and cultural narratives related to vaccine hesitancy.

**Results:**

The results indicate that from the perspective of health system stakeholders, opposition to measles vaccination is perceived as being primarily driven by deep-seated religious and socio-cultural convictions. Opposition is also driven by limited health literacy; EPCR 2024 data reveals that 34% of caregivers are unable to name a single vaccine in the routine program, and only 4% possess high vaccine knowledge. Key barriers identified include explicit theological rejection specific to Evangelical or Pentecostal worldviews (e.g., 'Jesus heals, not your vaccine'), often characterized by 'healing theology' which views vaccines as an interference with divine sovereignty. This is accompanied by a fundamental functional misconception of vaccines as curative treatments for the sick rather than preventive tools for the healthy, and ancestral skepticism regarding modern medicine’s impact on longevity. Additionally, institutional mistrust, fear of adverse side effects, and low educational levels among caregivers significantly contribute to resistance. Specific ethnic groups, such as the Antemoro and Antandroy, manifest localised social prohibitions against injections.

**Conclusions:**

Resistance to measles vaccination in Madagascar is multifaceted, rooted in fatalism, institutional mistrust, and functional misunderstandings of immunisation. To achieve national elimination goals, public health strategies must move beyond traditional biomedical solutions. Engaging local and religious leaders as trusted messengers is essential for building community trust and developing culturally sensitive communication strategies that integrate local realities into future vaccination campaigns.

**Supplementary Information:**

The online version contains supplementary material available at 10.1186/s12889-026-28130-5.

## Background

Measles remains a leading cause of infant mortality worldwide, despite the availability of a safe and effective vaccine [1]. Globally, an estimated 10.3 million cases occurred in 2023, representing a 20% increase compared to 2022, a surge primarily attributed to insufficient vaccination coverage [2]. In Madagascar, measles is a major public health concern, frequently causing deadly epidemics that affect young children [3]. While 80 confirmed cases were notified in 2022, the country experienced a severe outbreak in 2018 and 2019, which resulted in 244,650 reported cases and 1,080 deaths [4]. The 2024 Post-Measles Campaign Evaluation (EPCR) estimates national coverage at 75.3%, an improvement from the 65.3% recorded in 2022, but still notably below the 95% threshold required for disease elimination [3, 5].

To address this burden, the Malagasy Ministry of Public Health, through the Expanded Programme on immunisation (EPI), has implemented routine systematic vaccination alongside periodic follow-up campaigns conducted every three years [6]. The national policy mandates two doses of the Measles Vaccine (MV), with the first administered at 9 months and the second between 15 and 18 months [7]. In accordance with this strategy, a nationwide follow-up campaign was conducted in October 2024, targeting more than four million children aged 9 to 59 months.

Despite sustained global efforts, the effectiveness of mass vaccination programmes is often compromised by complex non-medical factors [8]. Globally, vaccine hesitancy, which encompasses a range of behaviours from delayed acceptance to outright refusal, is recognized as one of the most significant threats to global health security [8]. International literature suggests that resistance to vaccination often stems from “*worldview conflicts*”, where theological doctrines, such as fatalism, or traditional prohibitions clash with biomedical paradigms [9]. In addition, the erosion of institutional trust and the persistence of localised socio-cultural belief systems are documented as major barriers that underlie health behaviours [10]. This mistrust is often rooted in the historical legacy of colonial medicine and the fact that Christianity, particularly Protestantism, has historically been intertwined with state formation and governing elites in Madagascar since the 19th-century Merina monarchy. While Christian institutions often became vehicles for modern education and medicine, indigenous systems, rooted in ancestral veneration, have periodically resisted these state-led interventions as symbols of external political authority. Consequently, vaccine resistance may reflect a historically informed skepticism toward state-led interventions rather than purely theological dissent. Such factors can lead to a fundamental misclassification of vaccines as curative rather than preventive tools. The effectiveness of mass vaccination programmes in Madagascar is also often hampered by deeply rooted religious and socio-cultural factors [10].

Madagascar exhibits a complex religious landscape: approximately 32% identify as Catholic, 34% as Protestant (which officially includes the Lutheran (FLM) church), while between 22 and 26% report no religious affiliation or follow indigenous systems [11]. The interaction between these diverse belief systems and biomedical paradigms shapes vaccine acceptance.

Understanding these non-medical barriers is essential for developing targeted and culturally sensitive communication and community mobilization strategies. While geographic accessibility and logistical challenges remain relevant, the cultural fabric of Malagasy communities plays a decisive role in vaccine acceptance. The main objective of this article is to qualitatively explore the socio-cultural and religious beliefs and perceptions that act as barriers to the acceptance of measles vaccination in Madagascar, drawing on data collected across all 23 regions as part of the 2024 PMCE. By identifying specific reasons for non-vaccination, this study aims to formulate recommendations to improve the performance of future immunisation efforts and achieve national measles elimination goals.

## Methods

### Study site and context

The study was conducted across the entire territory of Madagascar, encompassing all 23 regions to capture the diversity of local dynamics and socio-cultural beliefs regarding vaccination acceptance. This extensive geographical scope was essential to integrate perspectives from various ethnic and regional contexts into the analysis of barriers to immunisation. The research was integrated into the qualitative component of the October 2024 Post-Measles Campaign Evaluation (PMCE), which followed a nationwide follow-up vaccination campaign targeting more than four million children.

### Study design

This research employed a qualitative and exploratory study design to gain an in-depth understanding of individuals' interpretations, experiences, and specific perspectives regarding measles vaccination. This approach allowed for a detailed exploration of non-medical factors, such as religious convictions and traditional prohibitions, which act as fundamental barriers to vaccine acceptance in the Malagasy context.

### Study population and sampling

The study population specifically targeted key stakeholders within the health system and the community who possessed firsthand knowledge of local vaccination dynamics. Participants included health officials at the regional and district levels (EMAR/EMAD), community leaders, and Basic Health centre (CSB) managers. A purposive and targeted sampling methodology was utilized to ensure the inclusion of informants with deep insights into community perceptions. Selection criteria prioritized individuals directly involved in the execution or evaluation of the October 2024 campaign to gather rich accounts of the challenges encountered and reasons for non-vaccination.

### Data collection tools and procedures

Qualitative data were collected through in-depth individual interviews conducted between December 2024 and April 2025. This process relied on purposive sampling of key informants, including health officials at the regional and district levels (EMAR/EMAD), community leaders, and Basic Health Centre (CSB) managers across all 23 regions. Interviews were carried out by experienced investigative teams using semi-structured interview guides specifically developed for this study. We acknowledge that the interview guides included specific questions on religious reasons for refusal, which may have prompted respondents to frame community behaviours through a theological lens, potentially representing a methodological limitation. To ensure accuracy, all interviews were recorded using tablets equipped with dictaphones. These guides addressed several critical themes, including: coordination and micro-planning of the campaign; logistics and input management; communication and social mobilization strategies, including the identification of hesitant groups; specific reasons for vaccine refusal and socio-cultural barriers; management of Adverse Events Following immunisation (AEFI), data management, and medical waste and strengthening of the routine immunisation system.

### Data analysis

The qualitative data were analysed using a manual inductive thematic content analysis methodology. This process involved several systematic stages: transcription and translation of the recorded interviews, familiarization with the raw data, and the creation of formal codes to organise significant concepts. Themes were categorised as 'theological' when they invoked divine intervention or scripture, 'cultural' when linked to ancestral customs or ethnic taboos, and 'socio-political' when referencing the State or external authority. To ensure analytic rigor, two researchers independently coded a subset of transcripts (triangulation), and discrepancies were resolved through consensus to validate the inductive themes. Methodological transparency was ensured through the interpretation of themes and the inclusion of relevant verbatim quotes representing the perspectives of the participants. A Large Language Model was used to assist with language editing and structural formatting of the manuscript.

We acknowledge several limitations in this study. A primary limitation is our reliance on the second-order perceptions of health officials and leaders rather than direct community voices. While these stakeholders provide critical system-level insights, their accounts of caregiver motivations may reflect institutional narratives rather than first-person beliefs. Additionally, as noted in the data collection procedures, the interview guides included specific questions on religious reasons for refusal, which may have prompted respondents to frame community behaviours through a theological lens, potentially limiting the emergence of other perspectives.

### Ethical considerations

The study protocol received formal approval from the Ethical Evaluation Committee for Biomedical Research (Comité d’Évaluation Éthique pour la Recherche biomédicale—CEER) of the National Institute of Public and Community Health (Institut National de Santé Publique et Communautaire—INSPC) (Ref: 021/2024-CEERINSPC/IRB). Signed written informed consent was obtained from all participants prior to the interviews, ensuring voluntary participation and strict confidentiality. All data were anonymised to protect the identity of the respondents.

## Results

The qualitative results demonstrate that opposition to measles vaccination in Madagascar is primarily driven by deep-seated religious and socio-cultural convictions, often reinforced by logistical gaps and a lack of trust in the health system.

### Barriers related to religious and socio-cultural beliefs

**Fig. 1:**
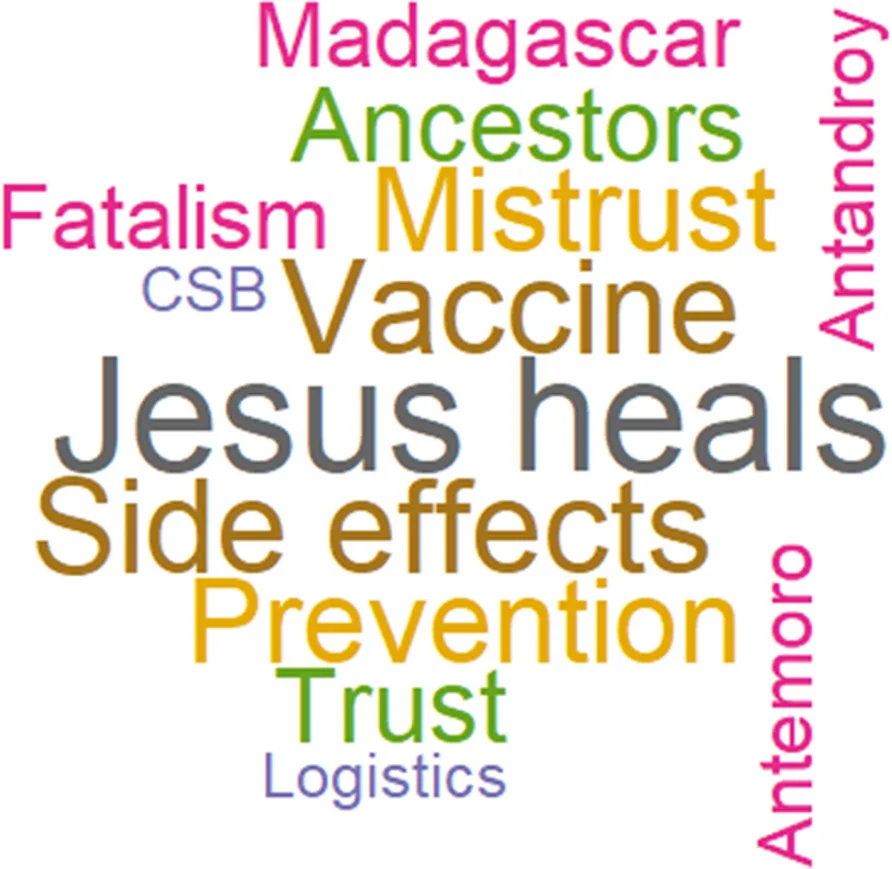
Word cloud of socio-cultural and religious barriers to measles vaccination in Madagascar

Qualitative interviews across the 23 regions revealed that spiritual and traditional frameworks often supersede medical recommendations (see Fig. 1).

- Religious theological opposition: A major barrier is the explicit rejection of vaccination by certain evangelical groups on spiritual grounds. This is best illustrated by a recurring verbatim: «*Jesus heals, not your vaccine*». Additionally, it was noted that «*households without religious affiliation were less exposed to campaign messages disseminated through the health centre and were less supportive of vaccination efforts*».
- The «*Treatment vs. Prevention*» misconception: A critical functional misunderstanding was identified: vaccines are perceived as a cure for the sick rather than a preventive tool for the healthy. Informants noted that parents often believe «*it is not worth giving an injection to healthy children*», agreeing to the shot only «*in cases of illness*». In some communities, traditional rituals precede medical seeking: «*they accept the injection in case of illness, but this, after having applied mud to the sick person… [some parents] applied mud to all the children and finally accepted the vaccination after conviction*».
- Ancestral skepticism and longevity narratives: Ethnic groups like the Antemoro and Antandroy express profound doubts about modern medicine by comparing it to ancestral lifespan. A key verbatim recorded was: «*They wonder why the ancestors lived up to 80–100 years while they didn’t get vaccinated? But today, people get vaccinated while their life expectancy is shorter? Thus, they said to themselves that the administration of vaccines kills people*».
- Institutional and ethnic mistrust: Specific communities, such as the «*Indian*» groups in urban areas, showed hostility due to a lack of trust in vaccination teams. It was reported that «*certain Indians refused to vaccinate their children due to the absence of trust in the vaccinators*». This was resolved by «*recruiting actors from their own community to be part of our teams, and it worked well*».

### Reasons for non-vaccination and structural barriers

Beyond beliefs, structural and educational factors create significant hurdles to full immunisation coverage. Economic barriers are significant: over 40% of the population avoids formal healthcare due to financial difficulties. During the campaign, transport costs remained a hurdle for 4.1% of families, particularly in urban areas (14.3%).

- Impact of education level: Multivariate analysis from the 2024 EPCR confirms that education is a primary discriminant: children of uneducated parents or those unable to read have a significantly higher risk (ORa = 2.19) of being 'zero-dose'. Caregivers with low educational attainment were identified as being more prone to vaccine hesitancy and influential in spreading skepticism. As one official stated: «*people with a low level of instruction… incite other members of the community to be reluctant*».
- Fear of Adverse Events (AEFI): Concerns regarding side effects represent a major psychological barrier, particularly for mothers. While men often focus on the vaccine’s perceived «*ineffectiveness*», women predominantly cite «*fear of side effects as their primary reason for opposition*». Furthermore, community workers sometimes avoid reporting these events: «*agents… do not dare or do not want to notify that certain children are victims of adverse effects… because they think that it happens because of the vaccination*».
- Communication and information gaps: Significant dissatisfaction was expressed regarding the timing and volume of information. While community agents served as the primary source of information for 79.7% of urban households, the reach of mass communication was often compromised by logistical and financial constraints. Problems included «*insufficient support: posters*», and «*negative criticism from certain groups*». Regarding media, it was noted: «*the spots announcements arrive with delay… instead of being broadcast a week in advance, the spots are broadcast only 3 to 2 days before the event*». These delays, frequently attributed to late funding and insufficient budgets for local launches, meant that essential messaging was only disseminated immediately before the campaign, exacerbating information gaps. In some districts, resources were very limited: «*we had to use the small speaker of the CSB*».
- Logistical challenges and access: Physical distance and the lack of personnel at sites were reported as frequent obstacles. One official noted: «*it is difficult for the teams working in remote sanitary sectors to return several times to the SDSP level to recover [vaccines]*». There were also financial concerns regarding motivation: «*GAVI was our partner… the payment is often late… this discourages some participants*».
- Household decision-making power: Gender and age dynamics within the family play a decisive role. It was highlighted that «*mothers were not authorised by the head of the household to vaccinate the child*». Permission is usually required from the «*head of the household*» or «*grandparents*».

### Health system stakeholders' perspectives

The coordination and micro-planning phases showed both strengths and critical weaknesses.

- Micro-planning Inconsistencies: Officials often felt that the central planning did not match local needs. A common complaint was that «modification of the validated microplan was noted after their return from the central level, leading to inconsistencies with the reality of the ground».
- Cold chain and infrastructure: Maintaining the vaccine quality was difficult in remote areas. Verbatim: «*the principal problem concerns the freezing… frequent electricity cuts… prevent the correct functioning of the batteries*». Officials suggested that «*drones would be an ideal solution*» for reaching enclaved districts.

## Discussion

Vaccine hesitancy, encompassing the spectrum of behaviours from delay in acceptance to outright refusal, represents a critical threat to global health security, especially regarding the resurgence of vaccine-preventable diseases like measles. This qualitative study demonstrates that opposition to measles vaccination in Madagascar is primarily driven by profound religious and socio-cultural convictions. Our results confirm that achieving high vaccination coverage requires addressing deep-seated cultural beliefs that underpin health behaviours rather than simply improving physical access [12, 13].

### Theological conflicts: «Jesus heals» and the doctrine of fatalism

A significant finding in the Malagasy data is the explicit opposition rooted in religious faith, particularly among certain evangelical groups stating: «*Jesus heals, not your vaccine*». This represents a «*worldview clash*» where medical intervention is viewed as irrelevant or inherently destructive compared to spiritual ethics. Such fatalistic belief systems, the conviction that disease outcomes are divinely predetermined, are documented drivers of negative vaccine attitudes globally [14, 15]. Our findings align with studies showing that individuals who believe disease is a matter of fate often exhibit the strongest resistance to modern immunisation efforts [16].

### Socio-cultural prohibitions and the erosion of institutional trust

Beyond generalized religious beliefs, specific socio-cultural norms act as profound local barriers in Madagascar. This study identified that ethnic groups like the Antemoro, coming mainly from the southeast of Madagascar and Antandroy mainly from the south maintain social prohibitions against injections, institutionalizing resistance within their communities. This underscores the necessity of moving beyond aggregated national data to address localised belief systems. Furthermore, the skepticism expressed through narratives of ancestral longevity, questioning why unvaccinated ancestors lived longer than modern vaccinated generations, reflects a deep-seated mistrust of government-led interventions. This institutional mistrust is a well-documented barrier in Africa, often rooted in the historical legacy of colonial medicine [9, 17]. Furthermore, these socio-cultural barriers are inextricably linked to structural socioeconomic determinants. While stakeholders frequently highlight religious dissent, the 2024 EPCR data indicates that over 40% of the Malagasy population renounces formal healthcare due to financial constraints. Consequently, what is perceived by officials as theological or cultural resistance may, in many cases, be reinforced by the material realities of poverty and the tangible costs of reaching vaccination sites, even when the service itself is provided free of charge. This suggests that 'hesitancy' is not merely a matter of belief, but is often compounded by limited household income and restricted access to services.

### The functional misconception: vaccines as treatment vs. prevention

A key qualitative revelation is the functional misconception that vaccines are a «cure» for the sick rather than a «*preventative tool*» for the healthy. Many parents agreed to shots only «*in cases of illness*», reflecting a fundamental miscategorisation of the vaccine's role. This behaviour often arises when the perceived risk of contracting the disease is low, diminishing the urgency for prophylactic immunisation [18]. While religious barriers in other contexts (such as Muslim-majority countries) often focus on vaccine composition (e.g., porcine gelatin) [19], the Malagasy context is unique in its focus on fatalism and the prevention-treatment paradox.

### Strategic imperatives: the essential role of trusted local messengers

Given that vaccine beliefs are influenced by deep cultural and spiritual factors, merely increasing vaccine availability is insufficient. The path forward for Madagascar mandates targeted and culturally sensitive communication [20]. Our study explicitly recommends the active involvement of local and religious leaders to build trust and bridge the gap between health goals and community beliefs [21]. This strategy is validated across Low- and Middle-Income Countries (LMICs), where faith-based figures are instrumental in mediating medical information [22, 23]. By recognizing the «*language of the unheard*» and embedding health strategies within the cultural fabric of Malagasy communities, the health system can more effectively overcome resistance and achieve measles elimination.

## Conclusion

Measles remains a significant public health threat in Madagascar, with its resurgence driven in large part by complex barriers to vaccine acceptance. This qualitative study successfully identified that opposition is multifaceted, including explicit theological rejection rooted in fatalism, profound institutional mistrust, and specific socio-cultural prohibitions against injections among identified ethnic subgroups. A major contributing factor to under-vaccination is the functional misconception of the vaccine as a treatment for illness rather than a necessary preventative measure for healthy children. To address these deep-seated convictions and achieve national elimination goals, public health strategies must move beyond traditional biomedical solutions and implement targeted, culturally sensitive communication. The active involvement of local and religious leaders is essential for building trust and bridging the gap between health goals and community beliefs, ensuring that cultural realities are fully integrated into future immunisation campaign designs.

## Supplementary Information

Additional file 1.

## Data Availability

The datasets used and/or analysed during the current study are available from the corresponding author on reasonable request.

## Abbreviations

CSB: Basic Health Centre
DPEV: Directorate of the Expanded programme on Immunisation
EMAD: District Management Teams (Équipes de Management du District)
EMAR: Regional Management Teams (Équipes de Management de la Région)
EPI: Expanded Programme on Immunisation
INSPC: National Institute of Public and Community Health
MV: Measles Vaccine
LMICs: Low- and Middle-Income Countries
PMCE: Post-Measles Campaign Evaluation
