# Supplementary material for: Health system stakeholders’ perceptions of religious and socio-cultural influences on measles vaccine hesitancy in Madagascar: a qualitative study
Source: BMC Public Health. 2026 Jun 15;26:2410. doi: 10.1186/s12889-026-28130-5 (PMC13495244; doi:10.1186/s12889-026-28130-5)
Supplement: Supplementary file 1 [file 12889_2026_28130_MOESM1_ESM.pdf]

Additional File 1: Semi-Structured Interview Guide (English version)

****

Study Title: Religious and socio-cultural beliefs as barriers to the acceptance of measles vaccination in Madagascar: A qualitative study

Target Population: Health officials (Regional/District - EMAR/EMAD), Community Leaders, and Basic Health Center (CSB) managers

****

Part 1: Coordination and Micro-planning

1. How was the October 2024 integrated campaign, encompassing measles, polio, and catch-up activities, coordinated at your level?
2. Were there any discrepancies between the centrally validated micro-plans and the operational realities, such as team numbers or targeted populations, you faced on the ground?
3. How did the implementation of a "hybrid strategy," involving different approaches for different vaccines, affect coordination and the workload of health staff?
4. How were local stakeholders, such as the Social Mobilization Committees (APART) and administrative authorities, involved in the planning and launch phases?

Part 2: Logistics and Input Management

1. Describe the challenges faced regarding the cold chain, specifically focusing on energy availability and the functioning of refrigeration equipment.
2. Were there difficulties in recovering or transporting vaccines and tools to remote sanitary sectors, and how were stock ruptures or the use of routine stocks managed?
3. What is your perspective on utilizing new technologies, such as drones or solar-powered equipment, to overcome geographic barriers in enclaved areas?

Part 3: Communication and Social Mobilization

1. What specific strategies were used to mobilize the community, and how did you collaborate with local youth associations, schools, or religious organizations?
2. How do you assess the timing and effectiveness of communication tools, including posters, radio spots, and banners, in relation to the campaign start date?
3. How did the community receive the campaign messages, and were there specific groups or urban minorities that were harder to reach?

4. What is your view on the benefits of starting communication earlier and utilizing local dialects to improve message uptake?

Part 4: Specific Reasons for Vaccine Refusal and Socio-Cultural Barriers

1. In your experience, what are the primary religious reasons families provide for refusing the measles vaccine?
2. Are there specific ethnic or socio-cultural prohibitions against injections that are prevalent in this region?
3. Do parents typically view the vaccine as a preventive tool for healthy children or as a curative treatment for the sick?
4. Have you observed traditional rituals being used as a prerequisite or an alternative to modern vaccination?
5. How do narratives comparing current lifespans to the longevity of unvaccinated ancestors affect trust in modern medicine?
6. Who typically holds the decision-making power regarding a child's vaccination within the household, and whose permission is required?

Part 5: Management of Adverse Events (MAPI) and Waste

1. How are side effects or adverse events reported and managed in your area?
2. Do you believe health agents avoid reporting adverse events due to fear of being blamed for a child's illness, and how does this affect data?
3. Describe the availability and quality of medical waste management tools, such as incinerators or secured pits, at the CSB level.

Part 6: Data Management and Routine System Strengthening

1. How was data collection handled using digital platforms, and what challenges were faced regarding updates, internet connectivity, or data duplicates?
2. How can the lessons and data from this mass campaign be used to identify and catch up with "zero-dose" children in the routine immunization system (EPI)?
3. What specific roles should local, religious, and traditional leaders play to ensure the long-term sustainability of routine vaccination?
